# Highly Sensitive Magnesium-Doped ZnO Nanorod pH Sensors Based on Electrolyte–Insulator–Semiconductor (EIS) Sensors

**DOI:** 10.3390/s21062110

**Published:** 2021-03-17

**Authors:** Ensaf Mohammed Al-Khalqi, Muhammad Azmi Abdul Hamid, Naif H. Al-Hardan, Lim Kar Keng

**Affiliations:** 1 Department of Applied Physics, Faculty of Science and Technology, Universiti Kebangsaan Malaysia (UKM), Bangi 43600, Selangor, Malaysia; ensafalkhalqi@tu.edu.ye (E.M.A.-K.); naifalhardan@ukm.edu.my (N.H.A.-H.); 2 Physics Department, Faculty of Applied Science, Thamar University, Dhamar 87246, Yemen; 3 Pusat Pengajian Citra Universiti, Universiti Kebangsaan Malaysia, Bangi 43600, Selangor, Malaysia; kk@ukm.edu.my

**Keywords:** electrolyte–insulator–semiconductor (EIS), hydrothermal method, pH detection, Mg-doped ZnO nanorods

## Abstract

For highly sensitive pH sensing, an electrolyte insulator semiconductor (EIS) device, based on ZnO nanorod-sensing membrane layers doped with magnesium, was proposed. ZnO nanorod samples prepared via a hydrothermal process with different Mg molar ratios (0–5%) were characterized to explore the impact of magnesium content on the structural and optical characteristics and sensing performance by X-ray diffraction analysis (XRD), atomic force microscopy (AFM), and photoluminescence (PL). The results indicated that the ZnO nanorods doped with 3% Mg had a high hydrogen ion sensitivity (83.77 mV/pH), linearity (96.06%), hysteresis (3 mV), and drift (0.218 mV/h) due to the improved crystalline quality and the surface hydroxyl group role of ZnO. In addition, the detection characteristics varied with the doping concentration and were suitable for developing biomedical detection applications with different detection elements.

## 1. Introduction

pH monitoring is essential for many applications, such as medical, biological, and chemical analyses [1,2,3]. Most commercially available pH sensors are expensive, large, and bulky, and are therefore not appropriate for a wide range of biological applications. The ability to identify response flows at different values of pH is of great importance in chemical and biological methods. Lately, the development of pH sensors with a high stability and sensitivity for various biochemical and biological applications has attracted much attention from researchers. Since Bergveld introduced ion-sensitive field-effect transistors (ISFET) in 1970, different types of sensors have been used to estimate pH as well as other biochemical solutions, such as extended-gate field-effect transistors (EGFETs) [4], ion-sensitive field-effect transistors (ISFETs) [5], light-addressable potentiometric sensors (LAPSs) [6], and electrolyte insulator semiconductors (EISs) [7]. Among various types of sensors, the EIS system is considered one of the most exciting systems for chemical and biological sensing. Due to its small mass and size, label-free process, rapid response time, potential for real-time and multiplexed measurements, and compatibility with micro- and nano-fabrication technologies, it holds great potential for use in large-scale fabrication at low costs [8]. EIS sensors have been developed in different biosensing applications in the environmental and pharmaceutical environments [9,10,11]. Several sensing metal oxide films are used for pH sensing applications, such as IGZO, ITO, Ta_2_O_5_, MgO, Al_2_O_3_/SiO_2_, TiO_2_, CuO, and ZnO [12,13,14,15,16,17,18,19]. The flat-band voltage of the EIS device changes depending on the ionic conditions of the tested solution. Thus, choosing a suitable sensing layer is essential for producing very sensitive and accurate bio-sensors [20]. ZnO has been widely used in sensor applications as bulk, thin, and thick films, but nanostructures such as nanowires, nanorods, tetrapods, nanotubes, nanospheres, etc., offer many unique characteristics and have considerable promise for obtaining faster responses and higher sensitivities [21]. The smaller dimensions of nanowires/nanorods result in integration with very large contact surfaces and strong binding with biological and chemical reagents, making them perfect candidates for constructing functional devices [22]. They have been used widely in different types of applications due to their easy fabrication methods, biocompatibility, chemical stability, and important optical features, and could be utilised as transducers in the construction of biosensors [23,24,25,26,27]. Therefore, ZnO has been proven to be a pH-sensitive gate insulator for different types of sensors, such as EGFET [28,29], ISFET [30], LAPS [31], and EIS [19,20,27,32,33]. Since electrical transport in ZnO has a significant impact on sensors’ sensitivity, monitoring the conductivity of ZnO is essential. An efficient method for adapting and controlling the electrical and optical characteristics of ZnO nanorods is doping with appropriate elements, which is critical for their commercial application [34]. Doping with metal ions such as Cd, Cu, Fe, Er, Mg, and Mn is generally used to adjust the conductivity of ZnO to meet different application requirements [35,36,37,38,39]. Due to the similar radius of Mg^2+^ (0.57 Å) to that of Zn^2+^ (0.60 Å), it was easy to integrate into ZnO lattices through substitution [40]. This means that no phase transformations or lattice distortions will occur due to replacing Zn with Mg. This minimizes or removes native defects from ZnO nanostructures with non-stoichiometric properties.

The sensing performance of ZnO thin films or nanostructures for pH detection have been devoted to investigating in a view research recently. To date, to the best of our knowledge, the influence of various molar ratios of Zn/Mg ranging from 0 to 5% on the pH-sensing performance of Mg-doped ZnO nanorods has not yet been reported. This study investigated the sensing performance of Mg-doped ZnO nanorods with various doping contents by controlling the growth conditions using the hydrothermal method to control the size and shape of the nanorods, which can improve the sensitivity of the fabricated sensors. This method is low-cost, easy to handle, and the rods can be grown at relatively low temperatures.

## 2. Materials and Methods

Undoped ZnO and ZnO nanorods doped with different Mg concentrations were synthesized on silicon substrates (n-type). An ultrasonic process was used to clean the substrates using acetone, methanol, ethanol, and deionized water (DI) to eliminate any organic residues, dust, and oil that might exist on the surface of the substrates. A ZnO seed layer of approximately 80 nm was deposited via DC reactive sputtering using a PVD 75 sputtering unit (Kurt J. Lesker). The seed layers were annealed at 500 °C to improve the seed layer’s crystalline structure with Lindberg/Blue M™ box furnaces (Thermo Scientific™, Waltham, MA, USA). ZnO nanorods were prepared over the seeded substrates via the hydrothermal process. In the undoped ZnO nanorods, equal molar ratios (50 mM) of zinc nitrate hexahydrate Zn(NO_3_)_2_•6H_2_O and hexamethylenetetramine CH_2_)_6_•N_4_ were used and dissolved in DI water and stirred for 1 h at room temperature. To prepare Mg-doped ZnO nanorods, zinc nitrate hexahydrate and magnesium nitrate hexahydrate Mg(NO_3_)_2_•6H_2_O were used with Zn/Mg molar ratios of 49.5:0.5, 49:1, 48.5:1.5, 48:2, and 47.5:2.5 (mmol:mmol), and the corresponding samples were designated as Mg(1%), Mg(2%), Mg(3%), Mg(4%), and Mg(5%), respectively. The prepared samples were arranged vertically in the mixture inside the oven for 3 h at 95 °C. Then, the samples were cooled to room temperature and cleaned with DI water for various times and dried in a conventional oven for 30 min at 100 °C. Silver metal film was evaporated on the substrates’ backside as a contact and annealed at 300 °C for 10 min in an N_2_ atmosphere to ensure that the devices had Ohmic contacts. The chemical cell was composed of Teflon, O-rings were used to isolate the sensing area, and the cells’ electrode was made of aluminium metal. An Ag/AgCl (3 M KCl) reference electrode was used as a reference electrode to maintain a constant potential of the cell. All the analyses were performed at room temperature with a 50% humidity in a dark metal gage to eliminate any interference from the surrounding environment. Standard buffer solutions in a pH range from 4 to 10 were purchased from Bendosen. The fabricated EIS sensors were immersed in reversed osmosis (RO) water for 12 h before the measurements to saturate the silanol group of the tested sensing membranes.

The structure and morphology of the samples were investigated using X-ray diffraction (XRD) (Bruker D8 ADVANCE, Billerica, Massachusetts, MA, USA) and field emission scanning electron microscopy (FESEM) (LEO SUPRA 55VP, Carl Zeiss, Jena, Germany) with energy-dispersive X-ray spectroscopy (EDX, Oxford Inca, Abingdon, UK) to determine the elemental compositions. Photoluminescence spectroscopy (PL) (Edinburgh Instruments FLS920, Livingston, UK) with a xenon lamp with a power of 450 W was used to conduct optical emission measurements. The surface roughness (RMS) was determined by applying an atomic force microscope (AFM) (NX-10, Park system, Korea). Capacitance–voltage (CV) measurements were obtained using an LCR6002 (GW Instek, New Taipei City, Taiwan). The device was linked to a personal computer via a serial RS232 port and controlled using LabView V8.5 software. For the C–V measurements, a low frequency of 100 Hz and an AC voltage with an amplitude of 50 mV were used to maintain the electrochemical equilibrium.

## 3. Results and Discussion

### 3.1. Structure, Morphology, and Optical Properties of the Mg-Doped ZnO Nanorods

**Figure 1:**
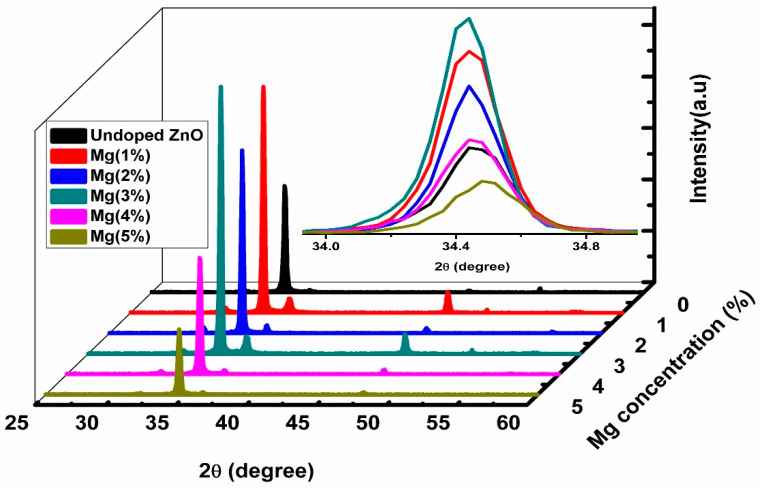
XRD patterns of the prepared undoped ZnO and Mg-doped ZnO nanorods via the hydrothermal method. The inset shows the shift in 2θ in the *c* axis (002).

The X-ray diffraction patterns of the undoped ZnO and Mg-doped ZnO nanorods samples are depicted in Figure 1. The XRD analysis confirmed that the patterns were consistently indexed to the standard diffraction patterns (JCPDS No. 36-1451) of a hexagonal ZnO structure, with a dominant (002) peak at a Bragg angle of approximately 34.50°. No Mg metal or Mg oxide peaks were found even when the concentration of Mg was increased to 5.0%, which indicates that the dopant was fully incorporated into the host lattice and the wurtzite structure of ZnO remains unaltered after Mg-doping [41,42]. The Mg substitution caused a slight shift in the Bragg angle’s position (34.47°–34.90°) as presented in the inset image in Figure 1, which might because of the Mg^2+^ dopant creating distortions in the ZnO lattice, producing crystal defects around the dopants [34,43]. The peaks’ intensity decreased as the Mg concentrations increased from 1 to 5%, except for Mg doped at 3%, which increased the most, confirming that this sample had the best crystallization quality and the Mg species occupied interstitial sites and substitution lattice sites [42,44]. The intensity ratio (I) of (002) peak to (101) peak reduced remarkably as Mg ions were substituted into the lattice of ZnO, confirming a larger growth rate on the (002) orientation in all samples as shown in Table 1. The crystallite size (*D*) of the (002) reflection peak was measured using Scherrer’s equation [19]:

(1) $$D=\;\frac{k\lambda }{\beta cos\theta },$$

where *k* = 0.9, *λ* is the wavelength equal to 1.54 Å, *β* is the full width at half maximum (FWHM), and *θ* is the half of Bragg’s angle in degrees. The crystallite size increased when the dopant concentration increased up to 3%, which corresponded to the optimal doping concentration (Mg (3%)) presented in Table 1. The same behavior was reported in the literature [45,46,47]. The crystallite size decreased as the Mg content increased to 4% and 5%, which was possibly because of the lower occupation of Mg in the ZnO lattice sites [48,49,50].

Doping generally causes imperfections in the crystallization of host materials in the form of defects and oxygen vacancies, which change the structure and geometric parameters of Mg-doped ZnO nanorods, as presented in Table 1. As can be noted in Table 1, the values of the lattice constants a and c were near the standard values for the hexagonal ZnO structure (a_0_ = 3.25 Å and c_0_ = 5.21 Å), and the c/a ratio proved that the prepared samples had nearly ideal wurtzite structures. The insignificant influence of the Mg contents on the lattice parameters of ZnO was probably because of the great solubility of the Mg ions in the lattice of ZnO [48]. The value of lattice strain showed an inverse trend in the grain size. The lowest strain value (7.91 × 10^−4^) at 3% Mg confirmed the high-quality epitaxial growth [51].

The number of defect states in the samples was estimated by the dislocation density formula [52]:

(2) $$\delta =\frac{1}{D2}.$$

The relationship between *δ* and *D* was inversely proportional, as displayed in Table 1. The wurtzite structure’s lattice parameters a and *c* were measured by the following expressions [42]:

(3) $$a=\frac{\lambda }{\sqrt{3}{\sin\theta }_{\left(100\right)}},$$

(4) $$c=\frac{\lambda }{Sin\theta_{\left(002\right)}}.$$

The lattice strain (*ε*) was used to measure the distribution of lattice constants arising from crystal imperfections in film, such as defects and lattice dislocations [40], and it was estimated by the equation [47]:

(5) $$\varepsilon =\frac{\beta cos\theta }{4}.$$

**Figure 2:**
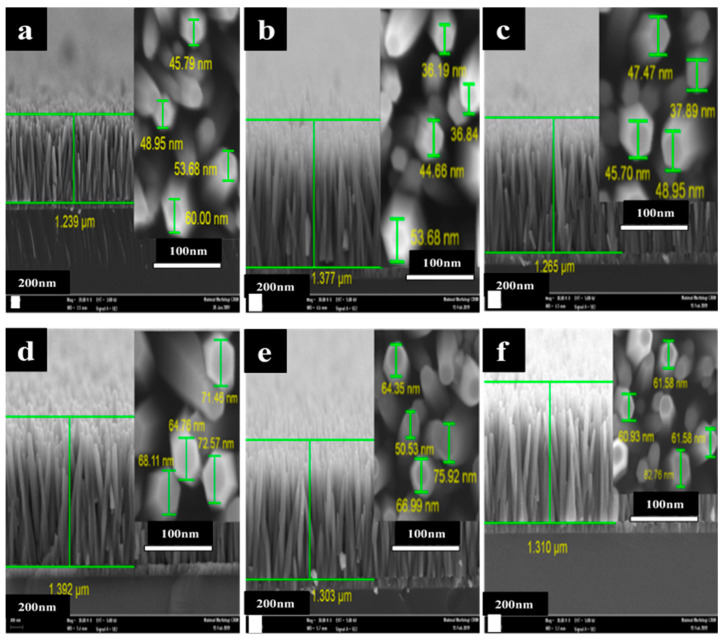
The FE-SEM cross section images of the ZnO nanorods doped with different concentrations of Mg: (**a**) undoped ZnO, (**b**) 1%, (**c**) 2%, (**d**) 3%, (**e**) 4%, and (**f**) 5%. The inset shows the surface view (the rod diameter).

The surface morphology, dimensionality, and density of the Mg-doped ZnO nanorods were investigated by FESEM. Figure 2a–f present cross-section and top-view images of the undoped ZnO and Mg-doped ZnO nanorods distributed throughout the whole area with a homogeneous density, smooth top surface, and highly vertical alignment along the *c* axis with a hexagonal structure. The ZnO seed layer had a strong impact on growing ideal rods, which can provide many advantages such as nucleation sites for growing ZnO nanorods, improving the films’ upper surface smoothness, uniform density, similar length, and vertical alignment along the *c* axis [53]. The average rod diameters increased with the Mg amount up to 3% with the higher contents of Mg, contrary to the length of the rods, which increased up to 3% from 1.24 to 1.39 μm then decreased to 1.30 and 1.31 μm, as presented in the inset image in Figure 2a–f. In Mg-doped ZnO nanorod samples, it was observed that the aspect ratio (length/diameter) was decreased by increasing the Mg concentration to 32, 27.7, 20.7, 20.2, and 19.6, which corresponded to Mg concentrations from 1% to 5%, which was the highest value compared to the values reported in the literature [54]. The rods’ shaped edges became clearer as the Mg content increased.

The EDX measurements were taken to illustrate the chemical composition of the undoped ZnO and Mg-doped ZnO nanorods samples prepared via hydrothermal process. Table 2 shows the contents of the elements at each molar ratio. The results are the average content of multiple spots over the surface of the prepared samples. Initially, the results indicated that the content of the prepared samples are Zn, O for the undoped ZnO and Zn, O and Mg for the doped ZnO nanorods. Increasing the molar ratio to 1, 2 and 3 shows an increase in contents of Mg corresponding to the molar ratio. However, further increasing in Mg contents to 4 and 5% show a suppress in the Mg contents. It was reported that the phase solubility of MgO in its bulk form stands at approximately 4 at% [55,56,57]. In the current study, however, it was found that the solid solubility of MgO in ZnO was 3%. The synthesis methods play a major role in the MgO solubility limit in ZnO [48]. The results of the current study were close to those of studies published by Abed et al. [46] and Al-Hardan et al. [48] for Mg-doped ZnO nanocrystals. It is also noted from the results in Table 2, that the actual quantity of Mg incorporated into the ZnO matrix is less than the nominal amounts (1%, 2% 3%, 4% and 5%), which may indicate the inhomogeneous distribution of Mg atoms on the surface of the samples. The results obtained in this study are similar to the work published by Hammad et al. [55] and Polat et al. [56], where they prepared Mg-doped ZnO via chemical deposition process.

**Figure 3:**
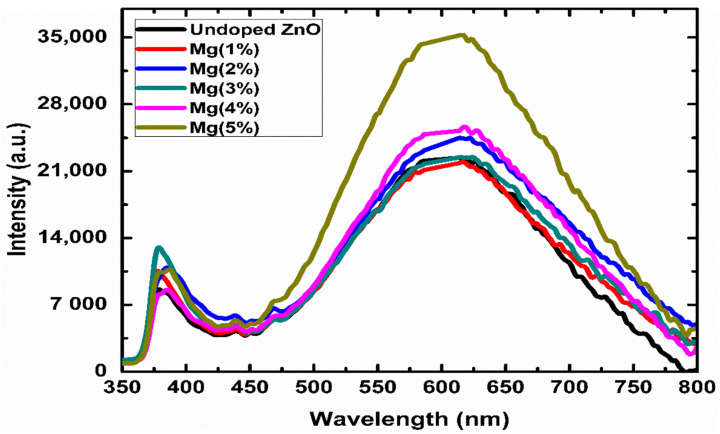
PL patterns of the ZnO and Mg-doped ZnO nanorods at room temperature at different molar ratios.

Photoluminescence (PL) analysis is a robust process for examining the impacts of impurity doping on the optical characterization of semiconductor nanostructures, since the optical features are expected to change after doping [34,45]. Figure 3 shows the room temperature PL spectra of the undoped ZnO-doped and Mg-doped ZnO nanorods. The excitation wavelength was 300 nm. In the visible spectra, two main emission peaks were observed—the near-band edge UV emission (~377 nm) and a broad emission band in the visible range (450–750 nm), which was centred around 600 nm in all the prepared samples. The near-band edge UV emission was attributed to the recombination of free excitons between the conduction and valence bands, while the broad emission band is usually attributed to the structural defects and impurities related to deep-level emissions [34].

As shown in the spectra presented in Figure 3, the UV peak intensities increased as the Mg concentration increased to 3%. The efficiency of the Mg-doped ZnO nanorods’ ultraviolet emission at various concentrations primarily depended on the crystalline quality. According to Bagnall et al. [57] the improvement in crystal quality was due to the reduction in impurities and oxygen vacancies, which cause a high near-band edge emission to deep-level emission ratio, leading to detectable UV emissions at room temperature. The greater the crystallization, the higher the density of free excitons and the greater the UV emission [58]. The increase ultraviolet emission was attributed to the improved crystalline quality as the doping concentration increased to 3%. This behavior was in accordance with stress variations in the Mg-doped ZnO nanorods. As the stress decreased (Table 1), the UV ratio increased, showing that low doping amounts reduced the defect density as Mg^2+^ filled the Zn vacancies. In addition, the surface of the ZnO grains was passivated by MgO, leading to a reduction in the density of the surface defects; consequently, the UV emission improved [48]. However, at Mg doping levels above 3% (4% and 5%), the increase in the stress was accompanied by a decrease in the UV ratio, indicating that excessive Mg (above 3%) induced structural defects. Similar behavior was reported in [45,46], and several groups reported enhancements in the UV bands’ intensity [42,44,59]. 

**Figure 4:**
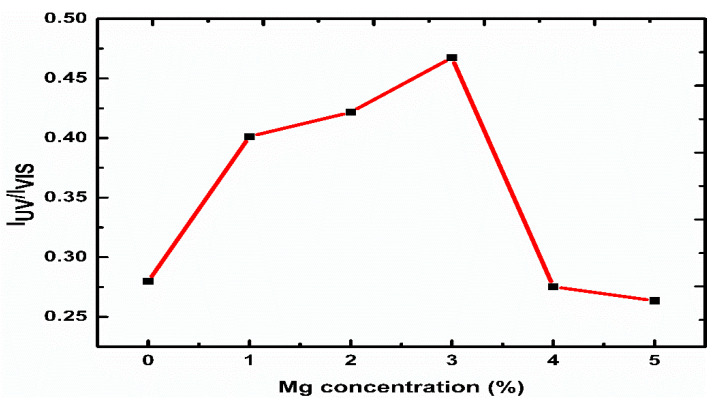
The intensity ratio I_UV_/I_VIS_ of the Mg-doped ZnO nanorods with different Mg concentrations.

The intensity ratio of the UV peak (I_UV_) to the visible peak (I_VIS_) as a function of the Mg contents is shown in Figure 4. This is considered one of the main factors that can be used to compare optical properties between samples. The ratio of I_UV_/I_VIS_ increased as the Mg amount increased up to 3%, and then decreased as the Mg content increased to 5%, which shows that the high UV improvement leading to the suppression of all surface defects of rods and decrease the separation of electron-hole combinations. The related results might have contributed to the remarkable increase in the visible emission (450–750 nm) compare to the UV emission for 4% and 5% Mg and hence the decrease in the I_UV_/I_VIS_ ratio [60].

**Figure 5:**
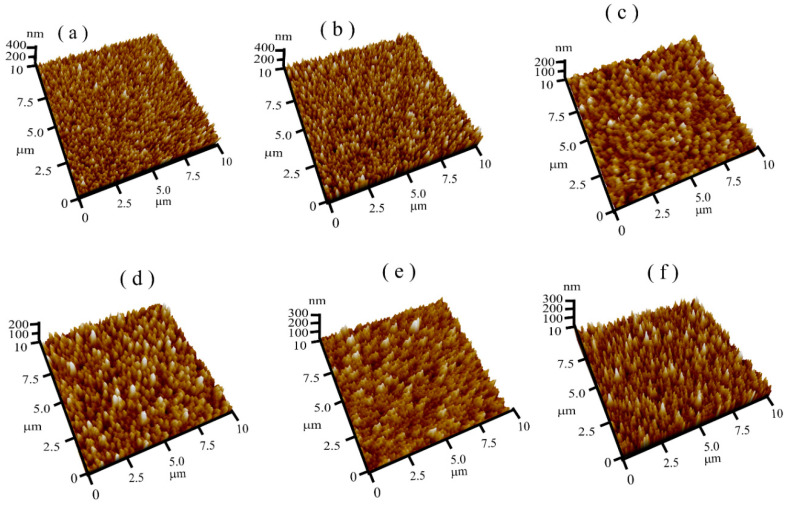
Three-dimensional AFM images of the ZnO nanorods doped with different Mg contents: (**a**) undoped ZnO, (**b**) 1%, (**c**) 2%, (**d**) 3%, (**e**) 4%, and (**f**) 5%.

**Figure 6:**
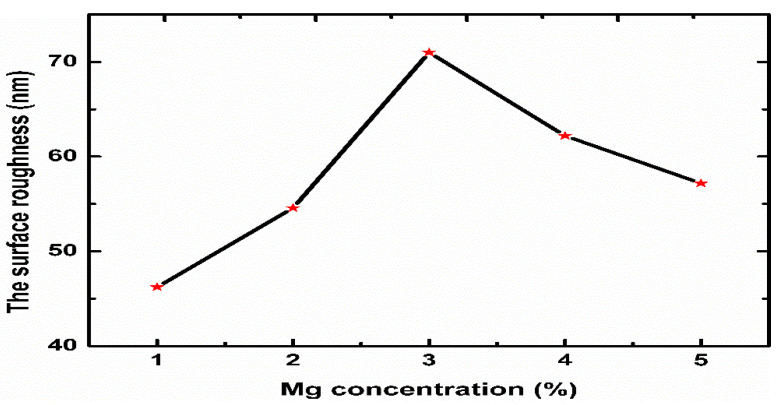
The surface roughness of the prepared ZnO nanorods as a function of the Mg concentration.

AFM was used to explore the surface morphologies and roughness of the Mg-doped ZnO nanorods. The changes in the morphology and the grain size play an essential role the enhancement of the pH sensitivity of EIS sensors [61]. Figure 5a–f show AFM images of the Mg-doped ZnO nanorods with different molar ratios ranging from 0 to 5%. As demonstrated by the AFM images, the Mg content had a significant effect on the ZnO nanorods’ surface roughness. The results indicated that, as the Mg content increased, the surfaces became rougher up to 3% (71 nm). However, further increasing the Mg content decreased the surface roughness to 62.20 and 57.18 nm in the samples doped with 4 and 5%, respectively. We believed that this behaviour was due to the increase in the self-diffusion of Zn, Mg, and O through an optimum doping Mg amount, improving the grain grouping [61] and thus increasing the surface roughness of the Mg-doped ZnO nanorods. Figure 6 displays the changes in the surface roughness of the Mg-doped ZnO nanorods as the Mg amount increased.

### 3.2. The Undoped ZnO and Mg-Doped ZnO Nanorod Sensing Performance

The capacitance–voltage (C–V) characteristics of the highly dense Mg-doped ZnO nanorod sensing membranes were studied to investigate the sensing performance of the Mg-doped ZnO nanorods towards pH buffer solutions in the range of 4–10 pH. C–V measurement is a suitable approach for measuring EIS devices and specifying system parameters, such as flat-band voltage, threshold voltage, Fermi level, and carrier density. These parameters’ values are considered as the base for other measurements and afford significant information depending on the potential electrolyte/insulator interference [20,62]. The main parameters which determinate the analytical characteristics of EIS are the sensitivity, selectivity, stability (drift), and hysteresis. The site-binding theory was first introduced by Yates et al. [63]. It is the most popular model used to characterize ionic (H^+^/OH^−^) absorption processes at electrolyte/oxide interfaces. The surface charge density is mainly related to the activity of ions in solution, and the density of different surface sites is acquired using various buffer solutions, which leads to different surface potentials. The changes in the potential values at the electrolyte/insulator interfaces was estimated using C–V measurements. As the pH values changed, the flat band voltage shifted. The corresponding voltages of all of the samples were calculated from the C-V curves with 0.5 C_max_ as a reference.

**Figure 7:**
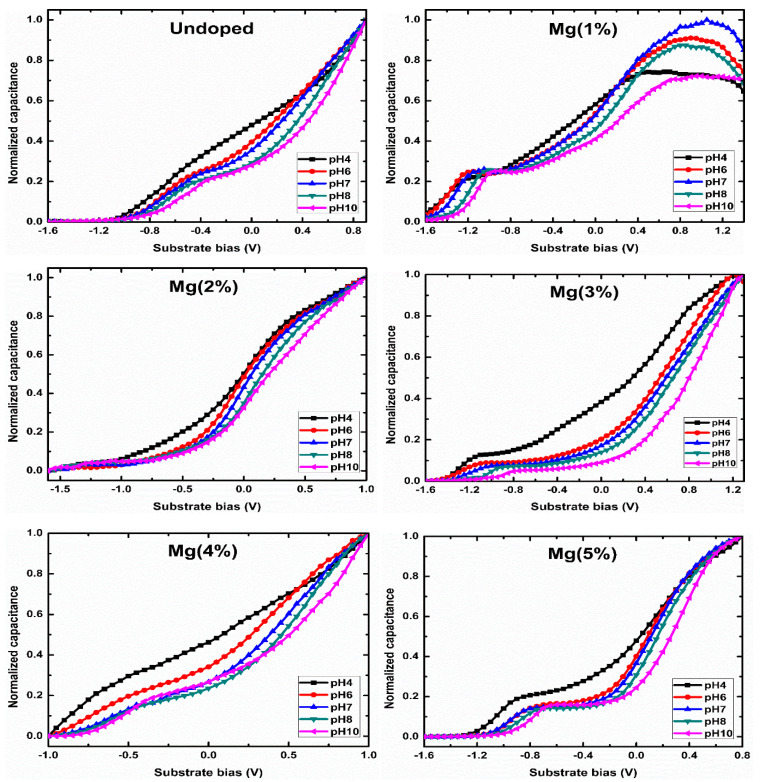
The normalized C–V curves of the ZnO nanorod samples undoped and doped with different Mg contents.

The following is the general expression for the sensitivity of the electrostatic potential of the electrolyte–insulator–semiconductor (EIS) system to changes in the bulk pH [64]:

(6) $$\frac{\delta \psi_{0}}{\delta pH_{S}}=-2.3\frac{kT}{q}\alpha ,$$

(7) $$\alpha =\frac{1}{\left(2.3kTC_{dif}/q^{2}B_{int}\right)+1}$$

where *k* is Boltzmann’s constant, *T* is temperature, and *q* is the elementary charge (*q* = 1.6 × 10^−19^ C); the dimensionless sensitivity parameter is  α, ranging between 0 and 1, according to the surface intrinsic buffer capacity$;\;B_{int}$ describes the capability of the oxide surface to transfer or take up protons; and $C_{dif}$ is the differential double-layer capacitance, which is essentially defined via the ion concentration of the bulk solution by the corresponding Debye length. From Equations (6) and (7), the maximum Nernstian factor (the sensitivity) of 59.3 mV/pH can be acquired only when *α* approaches 1 [64,65]. Figure 7 depicts the C–V curves of the Mg-doped ZnO nanorod sensing membranes under different pH values. 

**Figure 8:**
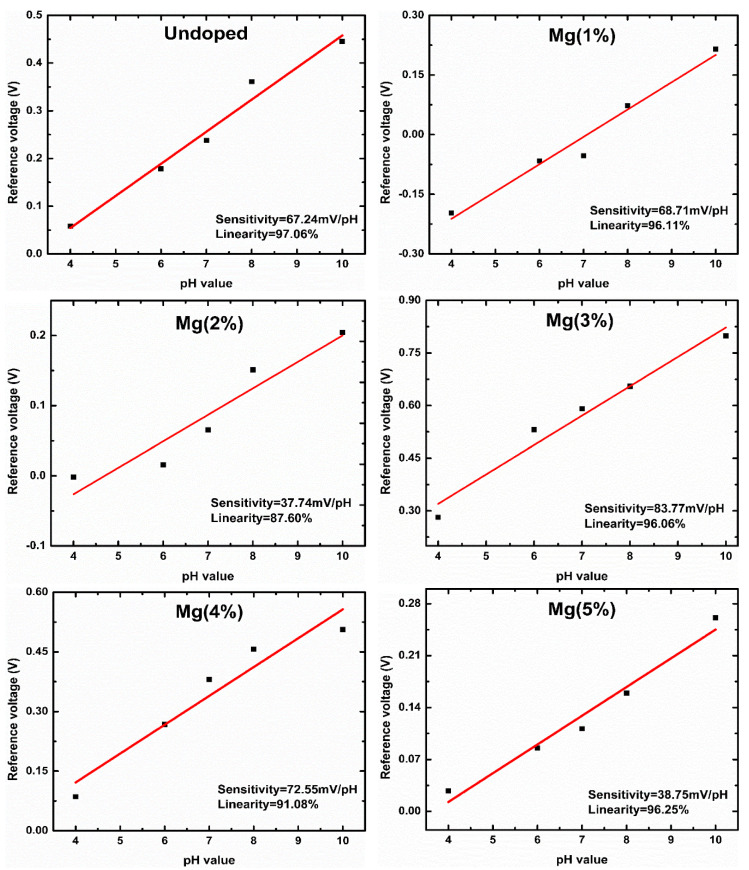
The pH sensitivity and linearity of the ZnO nanorods undoped and doped with different amounts of Mg.

The ZnO nanorods maximum threshold voltages shifted after the Mg doping reached 3%, showing that the devices’ sensitivity reached the highest value of 83.77 mV/pH with a 96.06% linearity. The undoped ZnO nanorod sensing membranes’ sensitivity was 67.24 mV/pH with a 97.06% linearity. The fabricated samples’ sensitivity after Mg doping was 68.71, 37.74, 83.77, 72.55, and 38.75 mV/pH at Mg contents ranging from 0 to 5%, respectively, as presented in Figure 8. The normalized C–V curves shift as the pH values increase, which is attributed to changes in oxide films’ capacity caused by changes in the number of binding sites available for H^+^ and OH^−^ on sensing membranes [33]. The incorporation of impurities improves the surface and interfacial material quality and increases the surface site density. Since Mg has a higher affinity to O, a doping process promotes the morphological changes and increases the crystal grain formation, supporting the increase in the surface roughness with the number of surface defects and improving the detection performance (the sensitivity and linearity) of devices [53,54].

A high surface roughness is essential for improving the sensing performance of fabricated EIS sensors. This is due to the increase in surface sites; consequently, further protonation and deprotonation will occur, increasing the potential at the oxide–electrolyte interface surface and resulting in higher sensitivity and linearity [61,66,67,68,69,70]. However, Mg ions can easily form because Mg has a low electronegativity (X = 1.31). Zn ions were substituted for Mg ions through the doping process, likely modifying the Zn-O bonds and increasing the surface density [71]. Several groups reported using EIS-based ZnO thin films as sensors to detect pH in various ranges, as shown in Table 3.

The results presented in Table 3 reveal that the sensing characteristics of EIS varied with the fabricating process used for the devices, such as annealing processes, NH_3_ plasma treatment, APTES immobilization, modifying the structures (such as nanorods and nanowires), and doping with suitable materials such as Ti and Mg, which all have numerous influences on the sensing performance of sensors.

**Figure 9:**
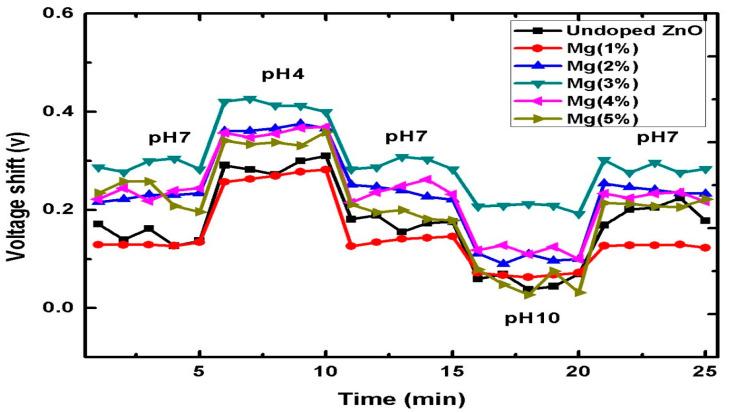
The hysteresis of the ZnO and Mg-doped ZnO nanorod sensing membranes.

The prepared samples hysteresis (short-term test) and drift (long-term test) were studied to evaluate their efficacy. To evaluate the hysteresis in the voltage output of the prepared EIS-based sensors, the undoped ZnO and Mg-doped ZnO sensors were tested in a loop of buffer solutions at different pH values (7 → 4 → 7 → 10 → 7) in an alternating time sequence, as shown in Figure 9. The hysteresis was calculated from the difference in the voltages between the initial pH 7 and the final pH7 in the pH loop [67]. The hysteresis of the undoped ZnO nanorods was 7 mV. The lowest hysteresis voltage was 3 mV for the 3% Mg-doped ZnO sample, and the highest hysteresis voltage was 17 mV in the 2% Mg-doped ZnO sample. Hysteresis is one of the parameters that decreases the reliability of EIS sensors [61]. This is mainly due to the interactions between the ions (H^+^ and OH^−^) present in the pH solution and the slow reactions of the buried sites of the membrane surface and/or the surface defects of the membrane [4,61]. According to Bousse et al. [72], Al-Hardan et al. [4] and Lin et al. [67], the diffusion of H^+^ ions into the buried sites of the sensing membrane is faster than the diffusion of the OH^−^ ions, the hysteresis is more important in an alkaline solution.

Drift is defined as the gradual change in the response of the sensors over time, while the pH value remains constant. The differences in the amount of surface sites over time are essentially due to the change in the chemical modification of the dielectric surface, consequently leading to an increase in the threshold voltage [73]. The difference in the reference voltage ($∆V_{ref}$) is given by:

(8) $$∆V_{ref}=V_{ref}\left(t\right)-V_{ref}\left(0\right).$$

**Figure 10:**
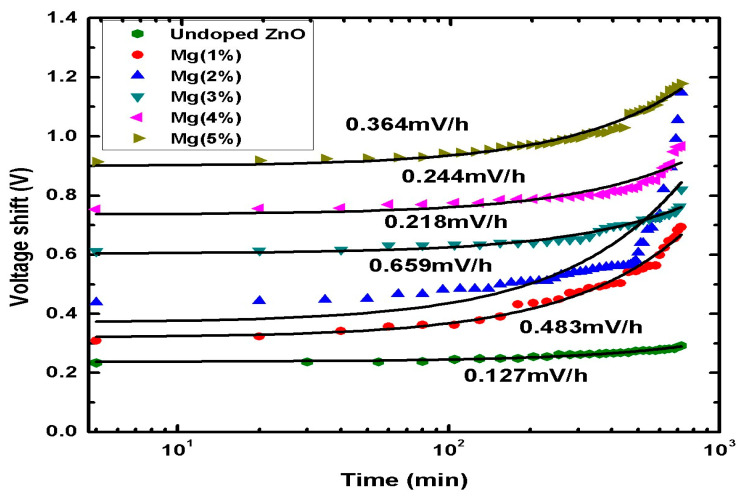
The drift rate of undoped ZnO and Mg-doped ZnO nanorod sensing membranes at different contents of Mg.

The change in the reference voltage might occur from lattice defects, which could be, for example, vacancies or dangling bonds caused by capturing groups of ions. These defects might be eliminated by controlling the parameters of the preparation method used for the sensing membranes, such as the annealing temperature [15,27,73] and the doping process [33,67], which result in the improvement of the drift voltage over time. In order to study the long-term stability (drift) of the sensing membranes, each sample was submerged in a solution of pH 7 for 12 h. Figure 10 presents the drift rates of the EIS devices based on Mg-doped ZnO nanorod sensing membranes doped at different contents (0–5%). Figure 10 shows that, among the samples doped with Mg, the EIS device with the 3% Mg-ZnO membrane exhibited the highest stability (0.218 mV/h), whereas the 2% Mg-ZnO membrane had the lowest stability of 0.659 mV/h. The highest sensing stability was observed in the 3% Mg-ZnO membrane, and it might have been due to the repair of defects in the Mg-doped ZnO nanorods resulting from the Mg incorporation. As a result, the extrinsic ions could neutralize the dangling bonds and compensate for the defects located underneath the insulator membrane for performance improvement [74]. The higher drift rate might be due to the high number of crystal defects [71].

## 4. Conclusions

In this study, we fabricated EIS sensors with Mg-doped ZnO nanorods sensing membranes prepared using a hydrothermal process for pH detecting and a super-Nernstian pH response were observed. The molar ratio of Zn/Mg significantly controls the crystal structure, morphology, and optical properties of the prepared samples. The EIS sensor with a 3 at% Mg-doped ZnO sensing membrane manifested an outstanding detection behaviour with a high sensitivity of 83.77 mV/pH. An insignificant hysteresis and a lower drift voltage were noted. In comparison with recently published results for EIS sensors based on ZnO thin films, the results of this study were superior.

## Figures and Tables

**Table 1:** Structural properties of undoped ZnO and Mg-doped ZnO nanorods with various doping concentrations.

Sample	2θ°	FWHM (2θ^o^)	Crystalline Size (nm)	δ × 10^14^	a (Å)	c (Å)	c/a	ε × 10^−4^	I(002)/(101)
Undoped ZnO	34.465	0.218	39.84	6.30	3.249	5.204	1.601	9.09	46.42
Mg (1%)	34.442	0.207	41.96	5.67	3.256	5.207	1.599	8.63	18.02
Mg (2%)	34.446	0.203	42.73	5.47	3.254	5.207	1.600	8.48	35.97
Mg (3%)	34.431	0.189	45.78	4.77	3.253	5.209	1.601	7.91	13.68
Mg (4%)	34.452	0.221	39.18	6.51	3.255	5.206	1.599	9.25	45.12
Mg (5%)	34.490	0.241	36.01	7.71	3.267	5.200	1.591	10.1	52.17

**Table 2:** The EDX spectra results of the undoped ZnO and Mg-doped ZnO samples.

Sample with Mg mmol:mmol	Zn (at%)	O (at%)	Mg (at%)
0.00	56.85 ± 0.26	43.15 ± 0.26	0.0
0.01	61.20 ± 2.26	38.70 ± 2.26	0.097 ± 0.01
0.02	56.58 ± 0.6	43.24 ± 0.66	0.19 ± 0.060
0.03	52.90 ± 0.71	46.85 ± 0.64	0.25 ± 0.071
0.04	54.78 ± 3.06	44.98 ± 2.96	0.25 ± 0.10
0.05	61.42 ± 3.0	38.32 ± 2.92	0.26 ± 0.13

**Table 3:** Comparison between the results of the fabricated EIS pH sensors based on Mg-doped ZnO nanorod sensing membranes with those of previous studies.

Sensing Membrane	Platform	Sensitivity (mV/pH)	References
ZnO	EIS	67.24	This study
Mg-ZnO at 1%		68.71	
Mg-ZnO at 2%		37.74	
Mg-ZnO at 3%		83.77	
Mg-ZnO at 4%		72.55	
Mg-ZnO at 5%		38.75	
ZnO	EIS	33.15	[27]
ZnO (600 °C)		42.54	
ZnO	EIS	31.20	[33]
ZnO (600 °C)		40.20	
Ti-doped ZnO		41.14	
Ti-doped ZnO (700 °C)		57.56	
APTES (3-aminopropyltriethoxysilane) functionalized ZnO nanorods	EIS	50.10	[32]
Mg-doped ZnO	EIS	38.32	[67]
Mg doped ZnO annealed at 700 °C		59.29	
ZnO	EIS	31.20	[71]
ZnO + NH_3_ (plasma 3 min)		47.02	
Mg doped ZnO		37.12	
Mg doped ZnO + NH_3_ (plasma 3 min)		53.82	

## Data Availability

Not available.
